# Supplementary material for: Not Too Warm, Not Too Cold: Thermal Treatments to Slightly Warmer or Colder Conditions from Mother’s Origin Can Enhance Performance of Montane Butterfly Larvae
Source: Biology (Basel). 2022 Jun 15;11(6):915. doi: 10.3390/biology11060915 (PMC9219776; doi:10.3390/biology11060915)
Supplement: Supplementary file 1 [file biology-11-00915-s001.zip › biology-1725199-supplementary.pdf]

Supporting Information

Title: Not too warm, not too cold: thermal treatments to slightly warmer or colder conditions from mother's origin can enhance performance of montane butterfly larvae

Konstantina Zografou [1*], George C. Adamidis [1,2], Brent J. Sewall [3] and Andrea Grill [1,4]

1. Institute of Ecology and Evolution, University of Bern, Baltzerstrasse 6, CH-3012 Bern, Switzerland; adamidis@upatras.gr (G.C.A.); a.grill@univie.ac.at (A.G.)
2. Laboratory of Plant Physiology, Department of Biology, University of Patras, 26504 Patras, Greece
3. Department of Biology, Temple University, Philadelphia, PA, 19122, USA; bjsewall@temple.edu
4. Department of Evolutionary Biology, University of Vienna, Djerassiplatz 1, A-1030 Vienna, Austria

*Correspondence: konstantina.zografou@temple.edu

Table S1. Averaged temperature treatments per climatic chamber during the time of the experiments. Individual caterpillars were equally divided into the three climatic chambers of high, medium and low temperature treatments (corresponding to low, medium and high elevation zone) after hibernation and mass measurements were taken systematically every week.
Temperature treatments cycled diurnally between the average long-term minimum and maximum historical temperatures in each climatic chamber. In addition, they were adjusted in a weekly interval to reflect seasonal changes in temperature, starting on the third week of June. The day-night-rhythm of the illumination was also adjusted weekly according to averaged weekly day length for 2019 (week 1/2 15.5 h, week 3 15.4 h, week 4 15.3, week 5 15.2 h, week 6 15 h, week 7 14.4 h, week 8 14.2 h). A gradual transition of one hour was applied between day and night conditions (and vice versa). The averaged values that included the day-night rhythm (photoperiod) were used in the analyses.

Treatments	Week	Day C°	Night C°	Averaged values with photoperiod
High C°	1	13.54	7.89	10.87
Medium C°	1	11.85	6.04	8.83
Low C°	1	8.79	3.51	6.14
High C°	2	14.53	8.69	11.70
Medium C°	2	12.71	6.69	9.63
Low C°	2	9.64	4.25	6.85
High C°	3	14.37	8.64	11.56
Medium C°	3	12.56	6.62	9.48
Low C°	3	9.55	4.08	6.73
High C°	4	13.77	7.98	10.90
Medium C°	4	11.82	6.09	8.87
Low C°	4	8.79	3.52	6.07
High C°	5	15.22	9.28	12.42
Medium C°	5	13.37	7.31	10.28
Low C°	5	11.04	5.32	8.14
High C°	6	14.77	9.29	12.01
Medium C°	6	13.06	7.37	10.11
Low C°	6	10.52	5.02	7.62
High C°	7	15.32	9.81	12.60
Medium C°	7	13.50	7.87	10.61
Low C°	7	10.66	5.35	7.88
High C°	8	13.59	8.26	10.96
Medium C°	8	12.10	6.54	9.21
Low C°	8	9.29	4.02	6.46

Table S2. Table accumulates all five *Erebia* butterflies and their offspring that participated in our experiment and were further considered in the statistical analysis. Mother's elevation is the elevation where the mother was originally collected in the field. Temperature treatment is the division of the individuals per species per elevation of origin into low, medium and high temperature treatment. Up/Down movements refer to upslope/ downslope movement from mother's origin elevation or temperature. In the last column an average of the weight is given for each individual in mg.

Species	MotherID	Individual	Mother's elevation	Mother's elevation (categorical)	Temperature treatment	Up/Down Movement	Average of Weight (gr)
Erebia aethiops	M2	In1	1823	Low	High	no change	0.96
Erebia aethiops	M2	In10	1823	Low	Medium	up1	0.43
Erebia aethiops	M2	In11	1823	Low	Low	up2	1.14
Erebia aethiops	M2	In12	1823	Low	Low	up2	0.88
Erebia aethiops	M2	In13	1823	Low	High	no change	0.86
Erebia aethiops	M2	In14	1823	Low	Low	up2	1.62
Erebia aethiops	M2	In15	1823	Low	High	no change	1.79
Erebia aethiops	M2	In16	1823	Low	High	no change	1.89
Erebia aethiops	M2	In17	1823	Low	High	no change	1.00
Erebia aethiops	M2	In18	1823	Low	High	no change	1.45
Erebia aethiops	M2	In19	1823	Low	Medium	up1	0.75
Erebia aethiops	M2	In2	1823	Low	Medium	up1	0.97
Erebia aethiops	M2	In3	1823	Low	Low	up2	0.85
Erebia aethiops	M2	In4	1823	Low	Medium	up1	1.54
Erebia aethiops	M2	In5	1823	Low	Low	up2	1.27
Erebia aethiops	M2	In6	1823	Low	Medium	up1	0.61
Erebia aethiops	M2	In7	1823	Low	Medium	up1	0.44
Erebia aethiops	M2	In8	1823	Low	Low	up2	0.73
Erebia aethiops	M2	In9	1823	Low	Medium	up1	0.59
Erebia aethiops	M5	In1	1823	Low	Medium	up1	1.73
Erebia aethiops	M7	In1	1823	Low	Low	up2	1.74
Erebia aethiops	MF3	In1	1735	Low	Low	up2	0.73
Erebia aethiops	MF3	In2	1735	Low	High	no change	1.51
Erebia cassioides	M11	In25	2123	Medium	Low	up1	1.53
Erebia cassioides	M11	In26	2123	Medium	Low	up1	0.87
Erebia cassioides	M11	In27	2123	Medium	Medium	no change	0.81
Erebia cassioides	M11	In28	2123	Medium	High	down1	1.35
Erebia cassioides	M11	In29	2123	Medium	Low	up1	0.57
Erebia cassioides	M11	In30	2123	Medium	Medium	no change	0.82
Erebia cassioides	M11	In31	2123	Medium	Low	up1	0.72
Erebia cassioides	M11	In32	2123	Medium	Low	up1	0.74
Erebia cassioides	M11	In33	2123	Medium	Medium	no change	0.67

Erebia cassioides	M11	In34	2123	Medium	Medium	no change	1.14
Erebia cassioides	M11	In35	2123	Medium	High	down1	0.74
Erebia cassioides	M11	In36	2123	Medium	Medium	no change	0.90
Erebia cassioides	M11	In37	2123	Medium	High	down1	1.35
Erebia cassioides	M11	In38	2123	Medium	Low	up1	0.88
Erebia cassioides	M11	In39	2123	Medium	Medium	no change	0.87
Erebia cassioides	M11	In40	2123	Medium	Low	up1	0.67
Erebia cassioides	M11	In41	2123	Medium	Low	up1	0.64
Erebia cassioides	M11	In42	2123	Medium	High	down1	1.04
Erebia cassioides	M11	In43	2123	Medium	High	down1	1.07
Erebia cassioides	M11	In44	2123	Medium	Low	up1	0.62
Erebia cassioides	M11	In45	2123	Medium	Medium	no change	1.50
Erebia cassioides	M11	In46	2123	Medium	Medium	no change	1.17
Erebia cassioides	M11	In47	2123	Medium	High	down1	1.24
Erebia cassioides	M11	In48	2123	Medium	Low	up1	0.97
Erebia cassioides	M11	In49	2123	Medium	High	down1	1.39
Erebia cassioides	M11	In50	2123	Medium	High	down1	1.41
Erebia cassioides	M11	In51	2123	Medium	Medium	no change	1.29
Erebia cassioides	M11	In52	2123	Medium	High	down1	0.91
Erebia cassioides	M11	In53	2123	Medium	Medium	no change	2.02
Erebia cassioides	M11	In54	2123	Medium	Medium	no change	1.56
Erebia cassioides	M11	In55	2123	Medium	Low	up1	0.99
Erebia cassioides	M11	In56	2123	Medium	Low	up1	0.66
Erebia cassioides	M11	In57	2123	Medium	High	down1	1.23
Erebia cassioides	M11	In58	2123	Medium	High	down1	1.69
Erebia cassioides	Mx1973	In1	2300	Medium	Low	up1	0.80
Erebia cassioides	Mx1973	In2	2300	Medium	Medium	no change	1.03
Erebia cassioides	Mx1973	In3	2300	Medium	High	down1	0.82
Erebia cassioides	Mx2013	In1	2275	Medium	Low	up1	0.48
Erebia cassioides	Mx2013	In2	2275	Medium	High	down1	0.45
Erebia cassioides	Mx2027	In1	2250	Medium	High	down1	0.44
Erebia manto	Ma3	In1	2283	Medium	High	down1	0.46
Erebia manto	Ma3	In10	2283	Medium	High	down1	0.54
Erebia manto	Ma3	In11	2283	Medium	High	down1	0.63
Erebia manto	Ma3	In12	2283	Medium	High	down1	0.60
Erebia manto	Ma3	In13	2283	Medium	Low	up1	0.31
Erebia manto	Ma3	In14	2283	Medium	Low	up1	0.55
Erebia manto	Ma3	In15	2283	Medium	High	down1	0.38
Erebia manto	Ma3	In16	2283	Medium	Medium	no change	0.33
Erebia manto	Ma3	In17	2283	Medium	Medium	no change	0.36
Erebia manto	Ma3	In18	2283	Medium	Low	up1	0.33
Erebia manto	Ma3	In19	2283	Medium	High	down1	0.50
Erebia manto	Ma3	In2	2283	Medium	Medium	no change	0.39
Erebia manto	Ma3	In20	2283	Medium	High	down1	0.46

Erebia manto	Ma3	In21	2283	Medium	High	down1	0.52
Erebia manto	Ma3	In22	2283	Medium	High	down1	0.57
Erebia manto	Ma3	In23	2283	Medium	Medium	no change	0.41
Erebia manto	Ma3	In24	2283	Medium	Medium	no change	0.45
Erebia manto	Ma3	In25	2283	Medium	Low	up1	0.53
Erebia manto	Ma3	In26	2283	Medium	Low	up1	0.77
Erebia manto	Ma3	In27	2283	Medium	Low	up1	0.39
Erebia manto	Ma3	In28	2283	Medium	Medium	no change	0.40
Erebia manto	Ma3	In29	2283	Medium	Low	up1	0.42
Erebia manto	Ma3	In3	2283	Medium	Low	up1	0.53
Erebia manto	Ma3	In30	2283	Medium	Low	up1	0.37
Erebia manto	Ma3	In4	2283	Medium	Medium	no change	0.63
Erebia manto	Ma3	In5	2283	Medium	Medium	no change	0.58
Erebia manto	Ma3	In6	2283	Medium	Medium	no change	0.78
Erebia manto	Ma3	In7	2283	Medium	High	down1	0.59
Erebia manto	Ma3	In8	2283	Medium	Medium	no change	0.47
Erebia manto	Ma3	In9	2283	Medium	Low	up1	0.36
Erebia nivalis	Mx193	In1	2531	High	Low	no change	0.46
Erebia nivalis	Mx193	In2	2531	High	High	down2	0.50
Erebia nivalis	Mx193	In3	2531	High	Medium	down1	0.49
Erebia nivalis	Mz10	In1	2481	High	Medium	down1	0.54
Erebia nivalis	Mz10	In2	2481	High	High	down2	0.75
Erebia nivalis	Mz10	In3	2481	High	Medium	down1	0.65
Erebia nivalis	Mz10	In4	2481	High	Low	no change	0.28
Erebia nivalis	Mz10	In5	2481	High	Low	no change	0.80
Erebia nivalis	Mz10	In6	2481	High	High	down2	0.73
Erebia nivalis	Mz10	In7	2481	High	Low	no change	0.64
Erebia nivalis	Mz10	In8	2481	High	Medium	down1	0.53
Erebia nivalis	Mz10	In9	2481	High	Low	no change	0.42
Erebia nivalis	Mz10	In10	2481	High	Medium	down1	0.56
Erebia nivalis	Mz10	In11	2481	High	High	down2	0.46
Erebia nivalis	Mz16	In1	2652	High	Low	no change	0.27
Erebia nivalis	Mz16	In2	2652	High	Low	no change	0.76
Erebia nivalis	Mz16	In3	2652	High	High	down2	0.46
Erebia nivalis	Mz16	In4	2652	High	High	down2	0.64
Erebia nivalis	Mz16	In5	2652	High	Low	no change	0.97
Erebia nivalis	Mz16	In6	2652	High	Medium	down1	0.72
Erebia nivalis	Mz16	In7	2652	High	High	down2	0.55
Erebia nivalis	Mz16	In8	2652	High	Medium	down1	0.85
Erebia nivalis	Mz16	In9	2652	High	Medium	down1	0.80
Erebia nivalis	Mz16	In10	2652	High	Low	no change	0.98
Erebia tyndarus	Mx1957	In1	2486	High	Medium	down1	0.35
Erebia tyndarus	Mx1957	In10	2486	High	Medium	down1	0.75
Erebia tyndarus	Mx1957	In11	2486	High	Medium	down1	0.53

Erebia tyndarus	Mx1957	In12	2486	High	Low	no change	0.66
Erebia tyndarus	Mx1957	In13	2486	High	Low	no change	0.57
Erebia tyndarus	Mx1957	In14	2486	High	Medium	down1	0.60
Erebia tyndarus	Mx1957	In15	2486	High	Low	no change	0.35
Erebia tyndarus	Mx1957	In16	2486	High	High	down2	0.43
Erebia tyndarus	Mx1957	In17	2486	High	High	down2	0.39
Erebia tyndarus	Mx1957	In18	2486	High	High	down2	0.59
Erebia tyndarus	Mx1957	In19	2486	High	Medium	down1	0.64
Erebia tyndarus	Mx1957	In2	2486	High	High	down2	0.38
Erebia tyndarus	Mx1957	In3	2486	High	Low	no change	0.41
Erebia tyndarus	Mx1957	In4	2486	High	Medium	down1	0.48
Erebia tyndarus	Mx1957	In5	2486	High	High	down2	0.34
Erebia tyndarus	Mx1957	In6	2486	High	Low	no change	0.52
Erebia tyndarus	Mx1957	In7	2486	High	High	down2	0.46
Erebia tyndarus	Mx1957	In8	2486	High	High	down2	0.70
Erebia tyndarus	Mx1957	In9	2486	High	Low	no change	0.65
Erebia tyndarus	Mx1964	In1	2486	High	Medium	down1	0.41
Erebia tyndarus	Mx1964	In10	2486	High	Medium	down1	1.32
Erebia tyndarus	Mx1964	In11	2486	High	Low	no change	0.71
Erebia tyndarus	Mx1964	In12	2486	High	Low	no change	0.74
Erebia tyndarus	Mx1964	In2	2486	High	Low	no change	0.81
Erebia tyndarus	Mx1964	In3	2486	High	High	down2	0.42
Erebia tyndarus	Mx1964	In4	2486	High	High	down2	0.67
Erebia tyndarus	Mx1964	In5	2486	High	Medium	down1	0.39
Erebia tyndarus	Mx1964	In6	2486	High	Low	no change	0.39
Erebia tyndarus	Mx1964	In7	2486	High	High	down2	0.76
Erebia tyndarus	Mx1964	In8	2486	High	Medium	down1	0.63
Erebia tyndarus	Mx1964	In9	2486	High	High	down2	0.92
Erebia tyndarus	Mx1965	In4	2486	High	High	down2	0.84
Erebia tyndarus	Mx1966	In1	2486	High	Low	no change	0.45
Erebia tyndarus	Mz7	In26	2274	Medium	Medium	no change	0.37
Erebia tyndarus	Mz7	In27	2274	Medium	High	down1	0.61
Erebia tyndarus	Mz7	In28	2274	Medium	Low	up1	0.47
Erebia tyndarus	Mz7	In29	2274	Medium	High	down1	0.60
Erebia tyndarus	Mz7	In30	2274	Medium	High	down1	0.93
Erebia tyndarus	Mz7	In31	2274	Medium	Medium	no change	0.41
Erebia tyndarus	Mz7	In32	2274	Medium	Low	up1	0.52
Erebia tyndarus	Mz7	In33	2274	Medium	Low	up1	0.55
Erebia tyndarus	Mz7	In34	2274	Medium	Low	up1	0.60
Erebia tyndarus	Mz7	In35	2274	Medium	Medium	no change	0.44
Erebia tyndarus	Mz7	In36	2274	Medium	Low	up1	0.47
Erebia tyndarus	Mz7	In37	2274	Medium	Medium	no change	0.57
Erebia tyndarus	Mz7	In38	2274	Medium	Low	up1	0.50
Erebia tyndarus	Mz7	In39	2274	Medium	Medium	no change	0.54

Erebia tyndarus	Mz7	In40	2274	Medium	High	down1	0.46
Erebia tyndarus	Mz7	In41	2274	Medium	Medium	no change	0.62
Erebia tyndarus	Mz7	In42	2274	Medium	Low	up1	0.48
Erebia tyndarus	Mz7	In43	2274	Medium	High	down1	0.60
Erebia tyndarus	Mz7	In44	2274	Medium	Medium	no change	0.62
Erebia tyndarus	Mz7	In45	2274	Medium	High	down1	0.58
Erebia tyndarus	Mz7	In46	2274	Medium	High	down1	0.45
Erebia tyndarus	Mz7	In47	2274	Medium	Medium	no change	0.58
Erebia tyndarus	Mz7	In48	2274	Medium	Medium	no change	0.81
Erebia tyndarus	Mz7	In49	2274	Medium	Low	up1	0.53
Erebia tyndarus	Mz7	In50	2274	Medium	High	down1	0.46
Erebia tyndarus	Mz7	In51	2274	Medium	High	down1	0.25
Erebia tyndarus	Mz7	In52	2274	Medium	High	down1	0.61
Erebia tyndarus	Mz7	In53	2274	Medium	Low	up1	0.42
Erebia tyndarus	Mz7	In54	2274	Medium	Low	up1	0.54
Erebia tyndarus	Mz7	In55	2274	Medium	Low	up1	0.37
Erebia tyndarus	Mz7	In56	2274	Medium	Medium	no change	0.73
Erebia tyndarus	Mz7	In57	2274	Medium	High	down1	0.45
Erebia tyndarus	Mz7	In58	2274	Medium	Medium	no change	0.59
Erebia tyndarus	Mz7	In59	2274	Medium	Low	up1	0.37
Erebia tyndarus	Mz7	In60	2274	Medium	High	down1	0.79
Erebia tyndarus	Mz7	In61	2274	Medium	Medium	no change	0.36
Erebia tyndarus	Mz7	In62	2274	Medium	Medium	no change	0.72
Erebia tyndarus	Mz7	In63	2274	Medium	Medium	no change	0.62
Erebia tyndarus	Mz7	In64	2274	Medium	High	down1	0.75
Erebia tyndarus	Mz7	In65	2274	Medium	Low	up1	0.50
Erebia tyndarus	Mz7	In66	2274	Medium	Low	up1	0.66
Erebia tyndarus	Mz7	In67	2274	Medium	High	down1	0.52
Erebia tyndarus	Mz7	In68	2274	Medium	Medium	no change	0.68
Erebia tyndarus	Mz7	In69	2274	Medium	Low	up1	0.69
Erebia tyndarus	Mz7	In70	2274	Medium	High	down1	1.07
Erebia tyndarus	Mz7	In71	2274	Medium	Low	up1	0.38
Erebia tyndarus	Mz7	In72	2274	Medium	Medium	no change	1.29
Erebia tyndarus	Mz7	In73	2274	Medium	Low	up1	0.65
Erebia tyndarus	Mz7	In74	2274	Medium	High	down1	0.65

Table S3. Model fitted to explain differences in caterpillars' weight under different temperature treatments. Individual was fitted as a random coefficient. All terms included in the maximal model structure are shown, together with their contribution to the final model which contained them. Terms retained in the minimal model are shown in **bold**. Results are shown for models fitted for individuals that had two or more biomass measurements. Response variable was logged transformed and standardized by subdividing weekly weights to the first measurement (first week) and then discarded the first measurements from the analysis.

Variable	χ^2	Df	*P*-value
**Time**	33.11	1	<0.001
**UpDown**	11.21	4	0.02
**Treatment**	9.12	2	0.01
**Mother Elevation**	14.01	1	<0.001
**Time:UpDown**	11.69	4	0.02
**Time:Treatment**	9.19	2	0.01
Species	9.21	4	0.06
Temperature	0.23	1	0.63
Species:Time	4.00	4	0.41
Mother Elevation:Treatment	4.78	2	0.09
Species:Temperature	5.21	4	0.27

":" represents an interaction between terms; "UpDown" refer to upslope/ downslope movements; Treatment refers to temperature treatments

Table S4. Individual models fitted to explain differences in species performance under different thermal treatments. Individual was fitted as a random coefficient. ":" represents an interaction between terms. Results are shown for models fitted for individuals that had two or more mass measurements.

	Variable	χ^2	Df	*P*-value
E. aethiops	Treatments	1.75	2	0.42
	Time	13.35	1	<0.001
	Treatment:Time	6.64	2	0.04
E. cassioides	Treatments	13.56	2	<0.001
	Time	45.44	1	<0.001
	Treatment:Time	30.54	2	<0.001
E. manto	Treatments	4.15	2	0.13
	Time	4.30	1	0.04
	Treatment:Time	1.88	2	0.39
E. nivalis	Treatments	14.54	2	<0.001
	Time	0.03	1	0.86
	Treatment:Time	1.03	2	0.60
E. tyndarus	Treatments	9.28	2	0.01
	Time	4.09	1	0.04
	Treatment:Time	1.04	2	0.59

":" represents an interaction between terms; Treatment refers to temperature treatments.
